# Strengths and weaknesses of EST-based prediction of tissue-specific alternative splicing

**DOI:** 10.1186/1471-2164-5-72

**Published:** 2004-09-28

**Authors:** Shobhit Gupta, Dorothea Zink, Bernhard Korn, Martin Vingron, Stefan A Haas

**Affiliations:** 1 Computational Molecular Biology, Max Planck Institute for Molecular Genetics, Ihnestr. 73, D-14195 Berlin – Germany; 2 German Resource Center for Genome Research, INF 580, 69120 Heidelberg – Germany

## Abstract

**Background:**

Alternative splicing contributes significantly to the complexity of the human transcriptome and proteome. Computational prediction of alternative splice isoforms are usually based on EST sequences that also allow to approximate the expression pattern of the related transcripts. However, the limited number of tissues represented in the EST data as well as the different cDNA construction protocols may influence the predictive capacity of ESTs to unravel tissue-specifically expressed transcripts.

**Methods:**

We predict tissue and tumor specific splice isoforms based on the genomic mapping (SpliceNest) of the EST consensus sequences and library annotation provided in the GeneNest database. We further ascertain the potentially rare tissue specific transcripts as the ones represented only by ESTs derived from normalized libraries. A subset of the predicted tissue and tumor specific isoforms are then validated via RT-PCR experiments over a spectrum of 40 tissue types.

**Results:**

Our strategy revealed 427 genes with at least one tissue specific transcript as well as 1120 genes showing tumor specific isoforms. While our experimental evaluation of computationally predicted tissue-specific isoforms revealed a high success rate in confirming the expression of these isoforms in the respective tissue, the strategy frequently failed to detect the expected restricted expression pattern. The analysis of putative lowly expressed transcripts using normalized cDNA libraries suggests that our ability to detect tissue-specific isoforms strongly depends on the expression level of the respective transcript as well as on the sensitivity of the experimental methods. Especially splice isoforms predicted to be disease-specific tend to represent transcripts that are expressed in a set of healthy tissues rather than novel isoforms.

**Conclusions:**

We propose to combine the computational prediction of alternative splice isoforms with experimental validation for efficient delineation of an accurate set of tissue-specific transcripts.

## Background

The large difference between cells from different tissues is the consequence of a complex regulatory machinery guiding the tissue specific expression of genes and their transcripts. Several genes have been described to exhibit differential splicing patterns for different tissues (E.g. *PDE1C *[1]; *IRF-3 *[2]) that result either in alternative proteins or affect the regulation of the respective gene product [3]. Due to the large number of genes generating alternative transcripts as well as by the complicated splicing machinery involving a large variety of different proteins, mis-splicing events are also frequently observed. Some of these artificial splice isoforms are already linked to specific diseases like Hemophilia A, Marfan syndrome etc. [4,5].

The resource mainly used to predict tissue-specific expression is the rapidly expanding repertoire of expressed sequence tags (ESTs) in the public databases representing a wide spectrum of tissue types. Unlike serial analysis of gene expression (SAGE) which mainly yields the tissue specific expression of genes [6], the EST data additionally allow the identification of alternatively spliced transcripts [7-11].

Besides the detection of the existence of alternative splice isoforms the tissue annotation of ESTs can be used for the computational prediction of the expression pattern of these transcripts where the tissue-wise count of transcript-specific ESTs with respect to a random background distribution defines an expression level [12-14]. Transcripts that are significantly over-represented by ESTs derived from a single tissue are usually defined as being tissue-specifically expressed. However, different cDNA construction protocols like normalization [15] include subtractive hybridization and PCR amplification steps introducing an artificial enrichment of ESTs from lowly abundant transcripts. The level of enrichment depends on the number of normalization/amplification steps performed, measured as Cot or Rot [16]. This inconsistency in the correlation of the number of ESTs observed for a transcript and its real expression level may affect the reliability of computational predictions of tissue-specifically expressed transcript. Since the EST-based prediction of expression patterns might already be error-prone because of the lack of sufficient numbers of EST sequences for each tissue this might be further complicated by different cDNA library protocols. Consequently, EST data related to normalized cDNA libraries are excluded from analysis in several computational approaches that aim at predicting tissue-specific expression [13,17]. Because of these uncertainties we combined our computational prediction of alternative splice variants and their expression pattern with experimental validation of these iso-forms via RT-PCR on 40 different tissue samples in order to evaluate the predictive potential of ESTs.

## Results

The EST-based prediction of alternative splice iso-forms revealed 427 genes each contributing at least one potential tissue-specifically expressed variant. These variants show specificity for 28 different tissue types, where brain, testis and placenta account for approximately half of these transcripts (see additional file 1). Many of these genes (n = 210) exhibit isoforms that were exclusively detected due to ESTs derived from normalized libraries. These form a significant fraction (p-value: 8e-19) of the total genes that show tissue specific transcripts, since the number of ESTs derived from normalized libraries (896,645) is only 30% the total EST count (3,084,576) in tissues for which tissue specific isoforms exist.

Out of the 20 isoforms tested experimentally (see additional file 3 for details of experiments), 15 isoforms could be successfully verified in some tissue (Table 1). The remaining five variants are either likely to resemble rare transcripts according to the respective library construction protocol, or as in case of a disease-specific isoform (Hs.272688), the appropriate tissue sample was not available for experimental testing. Only four of the isoforms predicted based on the basis of normalized libraries could be validated using the standard RT-PCR conditions. For five additional isoforms a more refined protocol had to be applied in order to detect bands of significant strength. More sensitive PCR conditions frequently revealed expression in more tissues indicating low expression of the isoforms in these tissues. These results show the tendency of normalized libraries to be enriched for low-abundant transcripts.

**Table 1:** RT-PCR validation results for tissue and disease-specific splice isoforms. The experiments are categorized into three groups viz. tissue specific isoforms predicted via ESTs related to non-normalized libraries (1 to 4), tissue specific isoforms predicted only via ESTs derived from normalized libraries (5 to 16) and disease-specific isoforms (17 to 20). For some of the variants represented by normalized libraries, standard PCR did not reveal the isoforms. However, five of these isoforms were detected using refined PCR conditions. The experiments frequently validated the isoforms and the tissue type, but the predicted specificity was rarely verified.

Isoform	Gene	Chr.	Unigene	EST Evidence	ESTs	Cycles	Isoform	Specificity	Comment (Most sensitive PCR)	Norm. Level
1	Unknown	11	Hs.112250	testis	3	39	+	+		
2	ISGF3G	14	Hs.1706	stomach	10	39	+	-	Ubiquitous	
3	MRPL42	12	Hs.112110	stomach-lymph	5	39	+	-	Ubiquitous	
4	SGN3	17	Hs.6076	testis	3	39	-	?		
5	PC326	13	Hs.279882	testis	9	39	+	+	testis [36]	Rot-5
6	LMO7	13	Hs.5978	brain	5	39	+	-	brain, testis, eye(?) [18]	
7	HRD1	8	Hs.274122	brain	3	39	+	-	brain, eye, thymus, salivary gland, kidney	
8	Unknown	1	Hs.24119	pancreas	4	39	+	-	approx. 10 tissues	Cot-20
9	BCLG	12	Hs.11962	testis	4	39,78	?,+	+,+		Cot-5
10	RBPMS	8	Hs.80248	placenta	4	39,78	?,+	-,-	Ubiquitous	
11	SCML1	X	Hs.109655	testis	12	39,78	?,+	+,-	approx. 6 tissues	Rot-5
12	WNK1	12	Hs.432900	kidney	3	39,78	?,+	+,-	Digestive system [28]	Cot-25
13	NY-CO-10	5	Hs.23557	testis	3	39,78	-,+	?,+		Cot-5
14	Unknown	11	Hs.169100	testis	3	39,78	-,-	?,?		Rot-5
15	Unknown	16	Hs.48396	breast	4	39,78	-,-	?,?		Cot-230
16	CIDE-A	18	Hs.249129	breast	4	39,78	-,-	?,?		Cot-230
17	KCNAB2	1	Hs.298184	tumor	29	39	+	-	Ubiquitous	
18	SNRP70	19	Hs.174051	stomach ascites	25/26	39	+	-	Ubiquitous	
19	RAB1	14	Hs.227327	tumor	39/95	39	+	-	fetal(kidney, thymus, liver, spleen), ovary [19]	
20	Unknown	7	Hs.272688	tumor	12	39,78	-,-	?,?	relevant tumor sample not in set	

**Figure 2:**
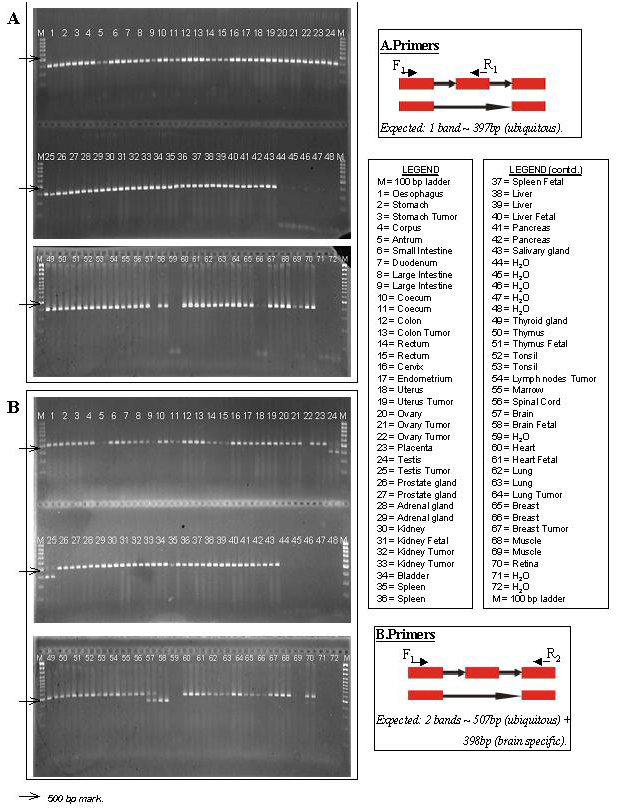
**RT-PCR validation experiment of a putative brain-specific isoform (*LMO7*)**. (A) The additional exon is detected in all tissues (primers F1, R1). (B) The primer pair F1-R2 located on exons flanking the extra exon results in two products where the shorter one is observed in brain, testis and eye (weak band). The predicted brain-specific expression pattern is, in fact, not specific.

The predicted expression of the isoforms in a single tissue could not be confirmed for half of the variants analyzed (standard conditions). However, the isoforms were always detected to be expressed in the tissue that was originally predicted by our software. The observed expression pattern of the 'unspecific' isoforms ranges from expression in only a few, sometimes related tissues (LMO7 [18]: brain, eye, testis, Fig. 2; HRD1: brain, eye, thymus, salivary gland, kidney) to ubiquitous expression (MRPL42, ISGF3G). Those variants that were validated to be specifically expressed frequently originate from testis. Increasing the sensitivity of the RT-PCR revealed another testis-specific variant. At the same time the variants of the genes WNK1 and SCML1 were no longer defined as being tissue-specifically expressed since they were now also detected in a few additional tissues (Table 1: isoform 11 & 12).

The number of genes with transcripts exclusively expressed in tumors is relatively large (1120) as compared to the number of genes revealing tissue specific isoforms. Interestingly, 2 out of 4 such disease-related transcripts (Table 1: isoform 17–20) were ubiquitously expressed although the large number of ESTs covering these variants was suggesting a high significance of the prediction. The tumor associated isoform described by Wang et al. [19] was observed to be expressed in several fetal tissues along with ovary.

The entire dataset for human as well as the gel images from the RT-PCR experiments is available at .

## Discussion

Consistent with previous work [11] our approach of combining computational and experimental validation yields a high success rate in predicting the existence of splice variants. In line with the expected general enrichment of clones derived from lowly expressed transcripts in normalized cDNA libraries our experimental results confirm the expression of the predicted low abundance transcripts. Consequently, those isoforms that could not be validated experimentally may also reflect real biological signatures of extremely rare transcripts since they are often represented just by heavily normalized libraries (Cot 230, CIDE-A + Hs.48396). While the methods used in the construction of normalized libraries (PCR amplification, subtraction, size selection) increase the sensitivity of the detection of transcripts they unfortunately disturb the rough correlation between the expression level of a transcript and the observed number of related clones that is usually maintained in non-normalized libraries. Therefore, in these cases, the larger number of ESTs found for a specific transcript will profess to deal with a higher expressed transcript, also implying a higher confidence in the prediction although the sequences may be derived from the same although amplified clone.

From the computational point of view the artificially increased number of ESTs affects the likelihood to predict tissue-specifically expressed transcripts since the prediction mainly relies on the count of ESTs [12,13]. Nevertheless, our experimental results show that especially isoforms predicted to be expressed exclusively in testis could be successfully validated, while other isoforms frequently appear to be expressed in a set of additional tissues that were not suggested by the ESTs. Surprisingly, the absence of supporting EST evidence for the variants LMO7 and ISGF3G is not caused by the lack of the respective cDNA libraries but may rather reflect differences in the tissue samples (e.g. enrichment of different cell types from the same organ, developmental differences) used for library construction.

In the context of tumors, our data shows that the predicted tumor-specific expression of isoforms derived from ESTs usually tends not to reflect the experimentally validated expression pattern. Rather it suggests expression in a collection of different tissues although the large number of related ESTs derived from tumor would imply a high confidence in the EST based prediction. Since tumor cells often show an up-regulation of a larger number of transcripts involved in various pathways [20,21] the tumor-specific transcripts predicted based on the EST data may just reflect this general deregulation of gene expression. The large number of predicted tumor-related isoforms further supports this hypothesis. Nevertheless, some transcripts detected via EST data may still serve as potential tumor markers like in case of the gene PRAME [22] where the EST data as well as the experimental data suggests specific expression in testis and in a variety of different tumors (see additional file 2).

Overall, ESTs are an extremely powerful tool to reliably unravel alternative transcripts independent of the level of expression. The functional relevance of the low abundant transcripts is not yet clear, especially if the isoforms do not affect the coding sequence. These isoforms may either be related to processes like nonsense-mediated decay (NMD: [23,24]) or they might be some kind of non-functional leakage of the splicing machinery. Nevertheless, since many lowly expressed genes are already known to have important regulatory functions [25-27] this may also hold true for a not yet defined fraction of the alternative isoforms we detected via normalized libraries. In contrast to the prediction of the existence of isoforms, the task of predicting their expression pattern is much more error-prone since EST data always covers only a subset of potential tissues with variable sensitivity. The fuzzy terminology of tissue-specific expression that is frequently used to describe significant expression in a discrete tissue or a set of tissues, is therefore strongly biased by the sensitivity of computational and experimental methods (SCML1; WNK1 [28]). Beside these technical difficulties, the definition of specificity may also depend on the regulatory network that mediates tissue-specificity. While isoforms expressed in testis are specifically expressed in a more strict sense, other isoforms are expressed in a small set of (not necessarily related) tissues eventually pointing to alternative regulatory mechanisms acting with different stringency, e.g. involving transcription factors [29], [30] and/or DNA methylation [31,32].

## Conclusions

The separate evaluation of EST data from non-normalized as well as from normalized cDNA libraries will help to categorize transcripts into highly and lowly abundant ones thus facilitating the integration of EST-based predictions with expression data from microarray experiments. We suggest that large-scale analysis of tissue-specific transcripts should be ideally based on a computational prediction of isoforms that ranks candidate transcripts, tightly coupled with experimental validation via RT-PCR or DNA microarray experiments [33]. Such an approach will lead to a comprehensive set of verified isoforms suitable for a wide range of applications in the functional analysis of the regulation of tissue-specific expression. This will also improve the detection of tumor-related isoforms that do not just reflect a general up-regulation of gene expression.

## Methods

The basis of our work is the tissue/tumor annotation of ESTs is GeneNest database [34] and the quality prediction of alternative splicing [11], visualized in SpliceNest database [10].

### Library classification

The cDNA libraries of the GeneNest database were semi-automatically categorized into non-normalized, normalized/subtracted and PCR-based libraries by screening for the appropriate keywords in the original annotation of the respective EMBL database entries. All libraries for which none of the keywords were found were defined as being non-normalized. PCR-based libraries like those derived by ORESTES PCR were not used for the current analysis. Additionally, to avoid miscounting caused by PCR amplification, ESTs of the same library and with identical start/end positions in the alignment were treated as a single sequence. Since the level of normalization of different libraries may differ depending on the number of rounds of subtractive hybridizations performed, we also extracted the normalization level (measured as Cot or Rot: [16]) as far as it was noted in the respective entries. Increasing Cot-values hereby reflect the enrichment of clones derived from low abundant transcripts in the respective cDNA library. Besides the categorization of cDNA libraries according to the construction methods used we further split these groups into libraries derived from healthy or disease tissue. Finally, ESTs of the four groups of cDNA libraries (healthy/non-normalized, healthy/normalized, disease/non-normalized, disease/normalized) were either analyzed separately or data of normalized and non-normalied libraries were combined.

### Prediction of tissue specific alternative splicing

**Figure 1:**
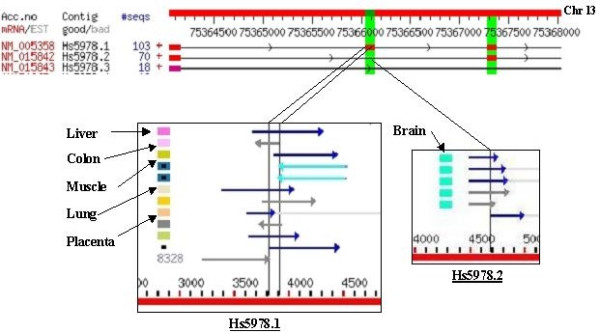
**Detection of brain specific splicing in gene *LMO7***. The top part of the figure is a visualization of gene *LMO7 *in SpliceNest, showing parts of three transcripts with exons displayed as red blocks, connected by lines representing introns. The middle exon of the top transcript (Hs5978.1) is missing in the second transcript (Hs5978.2) and is therefore highlighted as an alternative splice event (green bar). The boundaries corresponding to this exon as well as the corresponding intron are visualized as vertical lines in the GeneNest database (left and right box respectively). Both regions are covered by several ESTs depicted by horizontal arrows with corresponding tissues encoded in coloured rectangles towards the left of each EST. Upon comparing the tissue distribution of these alternative regions it is evident that the middle exon of transcript Hs5978.1 is covered by ESTs derived from several tissues, while the corresponding exon junction that lacks this middle exon, in transcript Hs5978.2, is represented by ESTs derived from brain only, thereby revealing this as a brain specific splice event.

Alternative splice isoforms in the SpliceNest database are revealed by aligning EST consensus sequences (putative transcripts) related to one gene to the appropriate genomic sequence. Significant differences in the boundaries of the putative exons are interpreted as alternative splicing events. For all exon-exon-boundaries that define a certain splice iso-form the annotation of ESTs covering the respective boundary is evaluated. Isoforms overrepresented by ESTs from particular tissue are tagged as putative tissue/tumor specific splice isoforms. Several parameters (e.g. number of ESTs from a particular tissue, number of ESTs from other tissues, number of associated mRNA sequences etc.) are computed for these isoforms and finally stored in a relational database system. The refined set of tissue and tumor specific variants is then generated by setting the requirement of at least 3 ESTs in both alternative forms. Fig. 1 describes such a prediction using GeneNest and SpliceNest visualizations. Since the counts of ESTs per tissue-specific splice event were frequently below 5, we considered it inappropriate to apply statistical methods as were used by Xu et. al. ([12]).

### Experimental verification

A set of putative tissue specific (n = 16) and disease-related (n = 4) alternative splice events was arbitrarily selected for RT-PCR experiments. PCR primers were generated on the alternatively spliced exon as well as on either side of the event (Fig. 2) using the primer design software GenomePRIDE ([35]).

For the subsequent RT-PCR experiment, total RNA was prepared using the single-step guanidinum method according to the manufacturer's instructions (TRIZOL, Gibco BRL). First strand cDNA synthesis was carried out in 20 μl reaction using the Omniscript Reverse transcriptase (Qiagen) and the oligo(dT) primers with 2 μg of total RNA. RT-PCR was carried out in a 20 μl reaction in 1 × buffer [1.5 mM Mg2+, 0.2 mM dNTPs, 0,4 μM primers each, 1 Unit of Taq polymerase (Roche)] and 1 μl of cDNA. Amplification steps were as follows: 95°C for 90 sec; 9 cycles of 94°C for 20 sec, 64°C for 10 sec decreasing the annealing temp for 1°C with each cycle (touchdown), 72°C for 20 sec; followed by 30 cycles of 94°C for 20 sec, 55°C for 10 sec, 72°C for 20 sec, followed by an extension at 72°C for 10 min. For the refined PCR, the amplification step was repeated with identical PCR conditions but with 2 μl of PCR product instead of 1 μl of cDNA.

All PCR products were resolved on 2% agarose gels run at 90 V/20 cm for 1.5 h in TAE buffer. Gels were then manually examined for exact size, genomic contamination and the tissues in which the bands are observed. As a control, a fraction of variants were sequenced using the ABI Prism BigDye Terminators and the ABI Prism 3100 sequencer (Applied Biosystems).

## Authors' contributions

SG wrote the prediction software as well as designed PCR primers for experimental analysis. SH and MV provided guidance for the computational work. DZ performed the RT-PCR experiments with the guidance of BK.

## Supplementary Material

Additional File 1 **List of tissues for which tissue specific transcripts are predicted. **This is a text file with a listing of all tissues for which specific trascripts exist along with the number of ESTs related to individual tissues. Furthermore, the ESTs derived from normalized libraries and the specific variants predicted via such ESTs are also listed. Click here for file

Additional File 3 **Detailed description of RT-PCR experiments **This is an excel file containing the primer sequences used for RT-PCR experiments along with detailed comments on the gel pictures subsequently obtained. Click here for file

Additional File 2 **RT-PCR picture (jpeg file) showing the expression pattern of gene PRAME **This gene shows specific expression for several tumor types, along with testis as the only normal tissue. Click here for file
