# Supplementary figures and images for: Strengths and weaknesses of EST-based prediction of tissue-specific alternative splicing
Source: BMC Genomics. 2004 Sep 28;5:72. doi: 10.1186/1471-2164-5-72 (PMC521684; doi:10.1186/1471-2164-5-72)

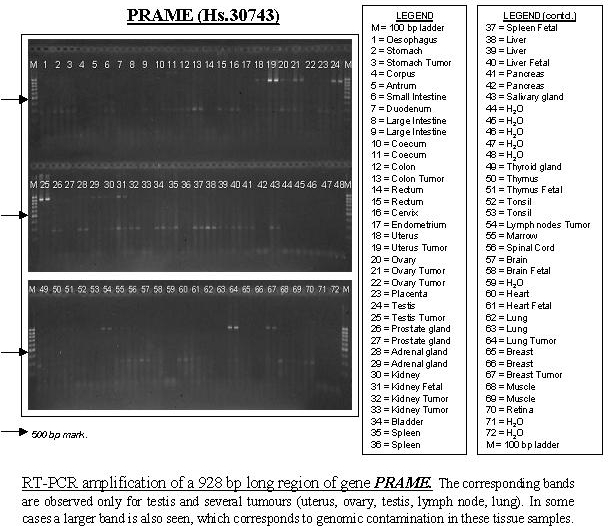

Supplement: Additional File 2 — RT-PCR picture (jpeg file) showing the expression pattern of gene PRAME This gene shows specific expression for several tumor types, along with testis as the only normal tissue. [file 1471-2164-5-72-S2.jpeg]
